# Aptamers for respiratory syncytial virus detection

**DOI:** 10.1038/srep42794

**Published:** 2017-02-21

**Authors:** Krisztina Percze, Zoltán Szakács, Éva Scholz, Judit András, Zsuzsanna Szeitner, Corné H. van den Kieboom, Gerben Ferwerda, Marien I. de Jonge, Róbert E. Gyurcsányi, Tamás Mészáros

**Affiliations:** 1 Department of Medical Chemistry, Molecular Biology and Pathobiochemistry, Semmelweis University, Budapest, Hungary; 2 MTA-BME “Lendület” Chemical Nanosensors Research Group, Department of Inorganic and Analytical Chemistry, Budapest University of Technology and Economics, Szt. Gellért tér 4, 1111, Budapest, Hungary; 3 Laboratory of Pediatric Infectious Diseases, Radboud Center for Infectious Diseases, Radboud University Medical Center, Nijmegen, The Netherlands; 4 MTA-BME Research Group for Technical Analytical Chemistry, Budapest University of Technology and Economics, Budapest, Hungary

## Abstract

The identification of the infectious agents is pivotal for appropriate care of patients with viral diseases. Current viral diagnostics rely on selective detection of viral nucleic acid or protein components. In general, detection of proteins rather than nucleic acids is technically more suitable for rapid tests. However, protein-based virus identification methods depend on antibodies limiting the practical applicability of these approaches. Aptamers rival antibodies in target selectivity and binding affinity, and excel in terms of robustness and cost of synthesis. Although aptamers have been generated for virus identification in laboratory settings, their introduction into routine virus diagnostics has not been realized, yet. Here, we demonstrate that the rationally designed SELEX protocol can be applied on whole virus to select aptamers, which can potentially be applied for viral diagnostics. This approach does not require purified virus protein or complicated virus purification. The presented data also illustrate that corroborating the functionality of aptamers with various approaches is essential to pinpoint the most appropriate aptamer amongst the panel of candidates obtained by the selection. Our protocol yielded aptamers capable of detecting respiratory syncytial virus (RSV), an important pathogen causing severe disease especially in young infants, at clinically relevant concentrations in complex matrices.

Respiratory syncytial virus (RSV) is a member of the Pneumovirus genus within the Paramyxoviridae family. The name of this enveloped virus derives from the visible consequence of the infection mechanism: merging the membranes of the nearby cells leading to the formation of the so called syncytia^1^. RSV is the most common viral agent of lower respiratory tract infections (LRTI) in young children. Almost all children encounter RSV infections before 2 years of age, which presents in the majority of cases with mild symptoms, comparable to the common cold, but a small portion of infants are at the highest risk, RSV is also a significant and often unrecognized cause of LRTI in both elderly and immunosuppressed patients^2^,^3^. The virus is very contagious and reinfection is a common phenomenon making RSV the most frequent cause of nosocomial transmission in pediatric wards^4^. Therefore, early and rapid diagnosis of RSV infection is imperative for immediate supportive care.

The traditional method of viral diagnostics, viral culture requires dedicated laboratories and its turnaround time does not fulfill the needs of rapid diagnosis. Presently, the most common RSV detection methods use either viral genome specific oligonucleotides for polymerase chain reaction (PCR) analysis or envelope protein selective antibodies for an immunoassay. Amongst the antigen detection approaches, the direct immunofluorescence staining (IF) and variations of the rapid antigen-based tests (RADTs) are most often used and both modalities are commercially available in ready-made kit format^5^,^6^. A potential way to increase shelf life and overall reliability of antigen detection approaches and their application in resource-limited locations is the replacement of antibodies with aptamers that are more resistant to thermal inactivation while featuring important theoretical advantages over antibodies^7^. Aptamers are short, single-stranded oligonucleotides selected *in vitro* in weeks that adopt unique conformations conferring selective binding of diverse target molecules^8^. Their ability of recognizing structural features of microbes is an emerging direction but with yet a vanishingly small panel of virus selective aptamers was isolated for diagnostic purposes.

Most laboratories applied the traditional SELEX (Systematic Evolution of Ligands by Exponential Enrichment) method for generation of virus selective aptamers, i.e. the selection was accomplished by using immobilized proteins^9^. However, some publications attested applicability of the variation of cell-SELEX, where the selection target was the inactivated virus particle^10^. Both approaches have their pros and cons. The protein demand of traditional SELEX is typically met by using protein expressing cell systems, which could provide ample amount of highly purified target protein that aids the oriented generation of selective aptamers for given viral proteins. However, most diagnostically important virus proteins are posttranslationally modified and the cell system produced proteins do not necessarily represent the native amino acid modifications^11^. Ironically, due to the high selectivity of aptamers, the application of inappropriately modified proteins as selection target (e.g. with a different glycosylation pattern) could result in aptamers without practical relevance since they cannot recognize viruses in clinical samples. Usage of whole viruses is expected to overcome this shortcoming of purified protein targets for aptamer selection, however it is severely limited by the complexity of virus purification. Still, given the difficulties of reproducing the glycosylation patterns of native viruses of clinical samples it projects as a more straightforward route for virus-selective aptamer selection. Additionally, using selective immobilization of viruses for the SELEX process to ensure the purity of the target as well as stringent counterselection steps against critical interferents may further improve the selection process.

Most of the aptamer related scientific publications report characterization of the selected oligonucleotides using a single methodology for interaction analysis and optimal buffers without interferents. Consequently, these studies provide very limited information about the practical applicability of selected aptamers. This common practice is likely to be one of the main limiting factor of the more extended leveraging of aptamers in diagnostics and therapeutics.

Here, we demonstrate that the rationally designed SELEX protocol using whole RSV particles selectively immobilized through a surface glycoprotein-binding monoclonal antibody and a counter-selection step with the most common co-infecting virus, results in selective aptamers with potentially diagnostic value. This approach, which could be generally applied for other viruses, will accelerate the process of selection as there is no need to use either purified viral protein or purified viruses. The presented data also highlights the importance of functional analysis of aptamer candidates by diverse approaches to pinpoint the most appropriate aptamers. Our protocol yielded an aptamer, which is capable of detecting RSV at clinically relevant concentrations in complex matrices.

## Results

### Selection of RSV discriminating aptamers

**Figure 1:**
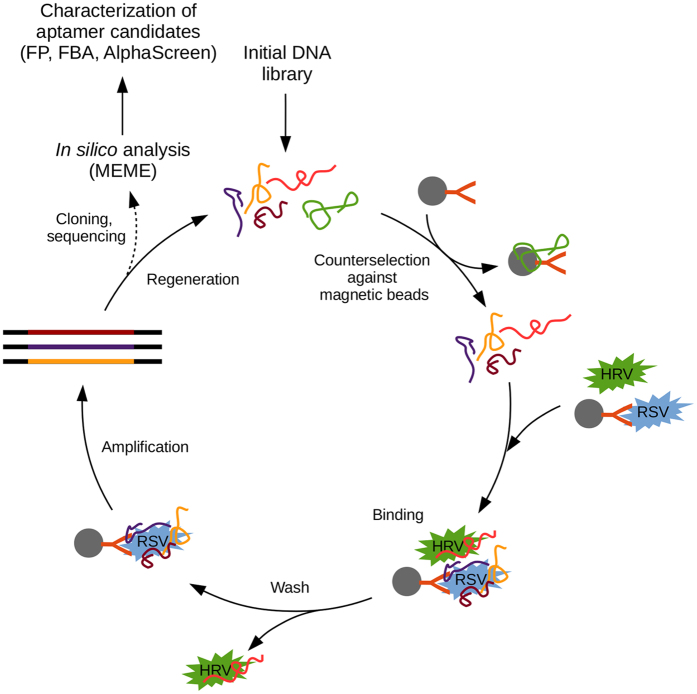
Flow chart of virus selective aptamer generation. See text for details. (FP, FBA and MEME stand for Fluorescence Polarization, Filter Binding Assay and Multiple Em for Motif Elicitation respectively).

Conventionally, the selectivity of generated aptamers is ensured by using a single molecule as target of selection. While the purity of this target molecule is essential as in most selective receptor generation methods^12^, an appealing advantage of SELEX is that implementation of counter-selection to eliminate oligonucleotide strands that bind critical interferents^13^ or even the whole sample matrix may alleviate this requirement. However, the best results are to be expected with the combined use of high purity target and counter-selection. Considering the difficulties and duration of virus protein purification, we decided to apply inactivated, partially purified whole virus for isolation of RSV discriminating aptamers. To comply with the purity requirements *in situ* purification was made by selective capturing of RSV using the F protein binding monoclonal Palivizumab antibody covalently linked to paramagnetic Protein A beads. Prior to the selection, a counter-selection was made to the immobilization matrix i.e., the Protein A beads and IgG binding aptamers were excluded by incubating the oligonucleotide library with antibody modified beads. To guarantee the specificity of isolated oligonucleotides, a second counter-selection was also introduced against human rhinovirus (HRV), a frequent coinfecting viral pathogen of the respiratory tract. Next, the immobilized RSV binding oligonucleotides were separated via four iterative cycles with gradually increasing selection pressure (Fig. 1).

**Figure 2:**
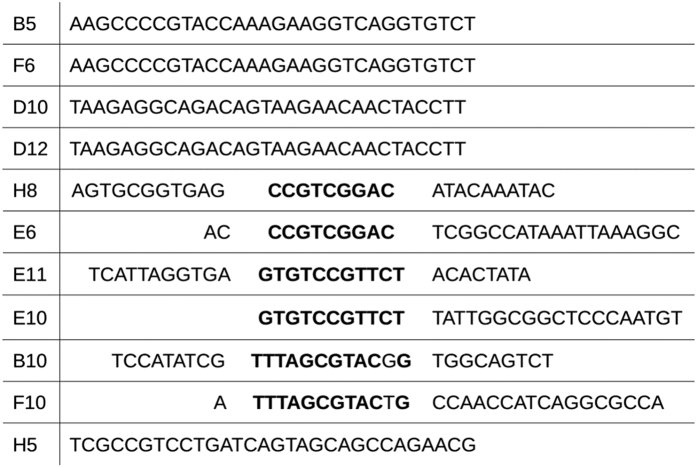
Nucleotide sequence of the studied aptamers. The sequence alignment of selected oligonucleotides resulted in identification of two aptamers in duplicated copies (B5, F6 and D10, D12); three pairs of aptamers holding an identical nucleotide sequence motif (H8, E6; E11, E10; B10, F10). The last studied aptamer was chosen randomly (H5) from the rest of the oligonucleotides that possessed unique sequences.

Following the last selection cycle, the PCR fragments were inserted into a cloning vector and the sequences of 96 colonies were determined by Sanger sequencing (See Supplementary Material Fig. 1). The MEME motif search revealed a sequence arrangement with 9 nucleotides^14^, which was present in two copies (H8 and E6) and other two motifs of higher E-values with 11 and 12 nucleotides (E11 and E10) and (B10 and F10), respectively (Fig. 2).

The alignment of sequencing data resulted in identification of two oligonucleotide pairs with identical sequence (B5, F6 and D10, D12) and the rest of the oligonucleotides possessed unique sequences. Further virus binding studies were restricted to these aptamers listed in Fig. 2.

### Functional analysis of aptamer candidates

Generally, the functionality of selected aptamers is investigated in optimal buffer conditions by using a single method; thus, the obtained results might be irrelevant considering the practical applicability of the analyzed oligonucleotide. To evade this shortcoming, we applied different approaches with complex protein matrices for identification of the most promising aptamer candidates.

**Figure 3:**
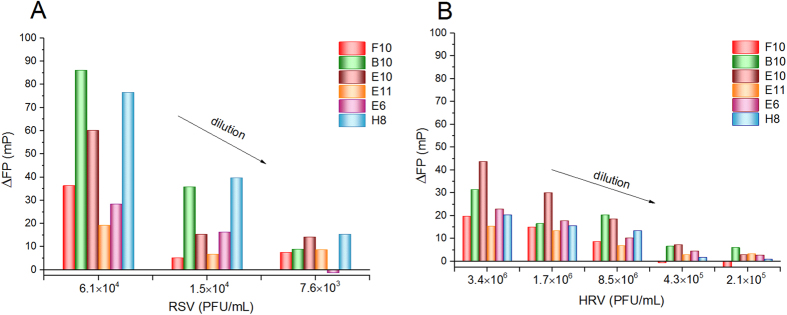
Fluorescence polarization change upon binding of various FAM-labeled aptamers (kept constant at 0.5 nM) to decreasing PFU RSV (**A**) and HRV (**B**) virus solution. Note that the HRV concentration in PFU equivalents is ca. two orders of magnitude higher than that of the RSV.

First, we studied virus binding capacity of consensus motif holding oligonucleotides in selection buffer by the conveniently implementable fluorescence polarization assay (Fig. 3).

A rapid screening of aptamers by fluorescence polarization confirmed binding for all the tested aptamers in a concentration dependent manner. However, the magnitude of the FP changes is not necessarily indicating the affinity of the aptamers to the targeted virus as the FP changes are also influenced by the extent in which the conformation of the aptamer to the virus impedes the free rotation of the FAM label. This may vary from aptamer to aptamer and therefore a simple comparison of the FP changes should be done with caution. With this in mind aptamers, E10, B10 and H8 seem to feature significantly better sensitivities for FP measurements. However, a screening against HRV shows the superiority of H8 from these three aptamers in terms of better discrimination of the HRV.

**Figure 4:**
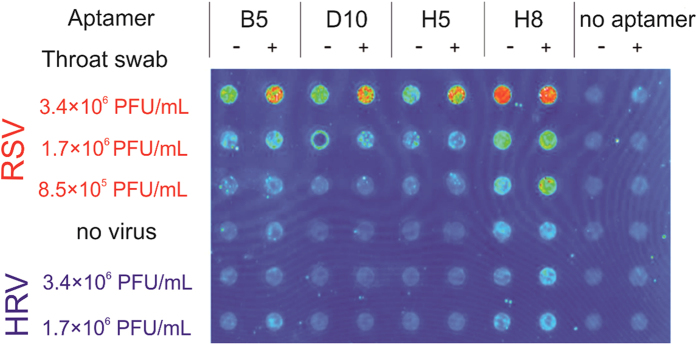
Functional analysis of aptamers by filter binding assay. FAM labelled aptamers were incubated with varying concentrations of RSV or HRV formulated in either PBS or throat swab completed PBS and vacuum blotted onto nitrocellulose membrane. The bound aptamers were detected by fluorescence imaging. The selective binding of all studied aptamers were indicated by the distinctively high signal of RSV spiked samples.

Next, we used the most traditional ligand binding characterization method, the filter binding assay^12^ as a simple and rapid functional test of sequenced oligonucleotides. The required fluorescently labeled aptamers were produced with PCR and alkali denaturation by using biotin and FAM modified primers. The created fluorescent aptamers represented the sequence found in duplicate (B5 and D10), the most promising conserved motif holding (H8), and a randomly chosen unique sequence (H5). The aptamers were kept at constant concentration and incubated with an incrementally decreasing number of inactivated RSV particles formulated either in PBS or in throat swab-based matrix. To assess the virus selectivity, the aptamers were also mixed with HRV containing phosphate buffer. Following the incubation, the mixtures were transferred onto nitrocellulose membrane and extensively washed before the fluorescence analysis. According to the obtained data, all aptamers selectively recognized RSV and produced low background signal with HRV samples. Of note, the aptamers remained selective even in the complex throat swab samples indicating their practical utility (Fig. 4). The highest fluorescence signal was detected by using the common motif holding oligonucleotide (H8), while the aptamers sequenced in duplicates (B5 and D10) produced the smallest intensity signal.

**Figure 5:**
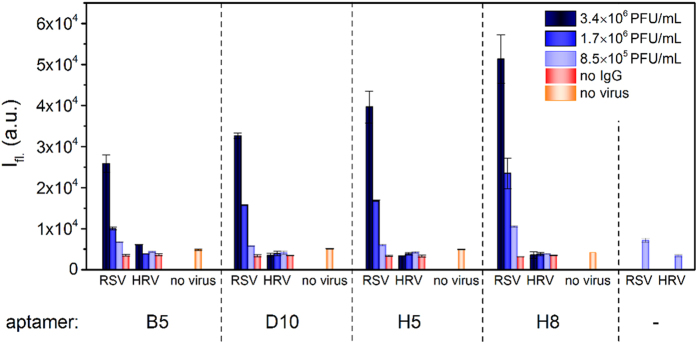
Functional analysis of aptamers by ALPHA. The biotin labelled aptamers were incubated with varying concentrations of RSV or HRV formulated in PBS. The reaction mixture was added to streptavidin coated donor beads and F protein selective antibody modified acceptor beads and the fluorescence intensities were measured.

Previously, we demonstrated the application of simple mix-and-measure Amplified Luminescent Proximity Homogenous Assay (ALPHA) for assessing the *K*_D_ of protein–aptamer complexes^15^. The approach relies on the use of donor and acceptor beads, which are coated with the binding partners. Here, we applied this approach using a RSV selective commercial antibody and the biotinylated oligonucleotides immobilized on the two type of beads that are brought in close proximity by binding to RSV. The donor bead contains a photosensitizer that upon excitation at 680 nm converts ambient oxygen to the singlet state, which diffuses and reacts with the thioxene derivative in the acceptor bead generating chemiluminescence. Due to the short lifetime of the singlet oxygen the luminescence indicates the close proximity of the beads, i.e. their binding to RSV. Here, we applied this approach using a RSV selective commercial antibody and the biotinylated oligonucleotides for interaction studies. The oligonucleotides were amplified using biotinylated forward and 5′ phosphate labeled reverse primers and treated with λ exonuclease to obtain single stranded aptamer candidates. Constant concentration of antibody and produced aptamers were mixed with varied amount of RSV or HRV. Next, the mixtures were completed with Protein A Acceptor Bead and Streptavidin Donor Bead and the interactions were determined by fluorescence measurement (Fig. 5). The data of ALPHA were in concert with the results of the filter binding assay; none of the four studied aptamer showed interaction with the HRV (no significant signal increase as compared with the virus-free control), but all bound RSV. Furthermore, as it was previously demonstrated by the filter binding assay, the consensus motif holding aptamer (H8) displayed the highest signal in this concentration range, while with the duplicate copy oligonucleotides (B5 and D10) the lowest signals were measured.

### Characterization of the virus protein specificity of the highest affinity aptamer

**Figure 6:**
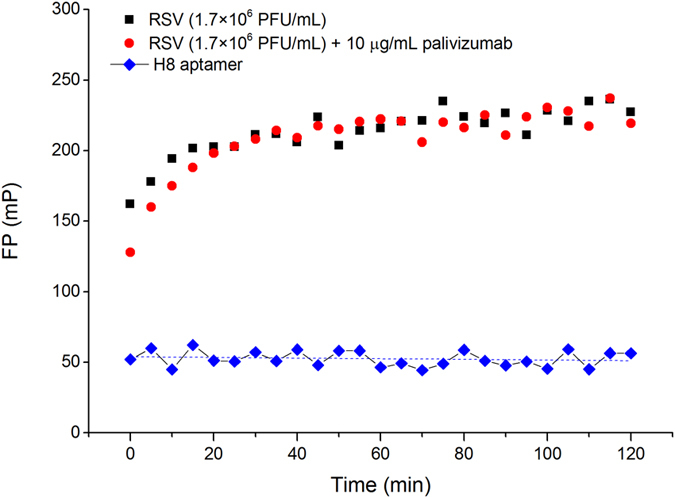
Aptamer (H8) binding to RSV and competitive effect of the F-protein selective palivizumab monitored by fluorescence polarization. The 0.5 nM Alexa Fluor 488 labelled H8 aptamer shows a polarization of ca. 50 mP that increases drastically in mixture with RSV indicating their binding. No change in the fluorescence polarization was obtained in the presence of a 100 × excess of F-protein selective antibody with respect of the aptamer.

The single-stranded, negative-sense RNA genome encodes 11 proteins that of which three are surface glycoproteins: the attachment protein G, fusion protein F, and the small hydrophobic SH protein^16^. The antibody used for the immobilization of the virus onto magnetic bead is known to recognize a conformational epitope of the fusion glycoprotein of RSV^17^. The fusion and attachment glycoproteins, two main proteins of RSV envelop are presented on the virion surface in many copies^16^. Consequently, the immobilized virions are expected to present available antibody binding epitopes during the selection procedure. To investigate whether the aptamer with the highest binding affinity (H8) and the antibody applied for immobilization during the selection (Palivizumab) compete for the same epitope, we set up an antibody-aptamer competition experiment using fluorescence polarization (FP). First, it was demonstrated that mixing the Alexa Fluor 488 labeled oligonucleotide with inactivated RSV resulted in substantial change in anisotropy proving the virus-aptamer interaction (Fig. 6). In the following measurement, the consensus motif possessing aptamer was mixed with two orders of magnitude higher concentration of Palivizumab antibody and then incubated with the RSV containing sample. The data did not show detectable fluorescence polarization changes upon addition of the large excess of antibody in comparison to the condition with aptamer only, indicating that the aptamer and the applied antibody did not recognize the same protein epitope (Fig. 6).

**Figure 7:**
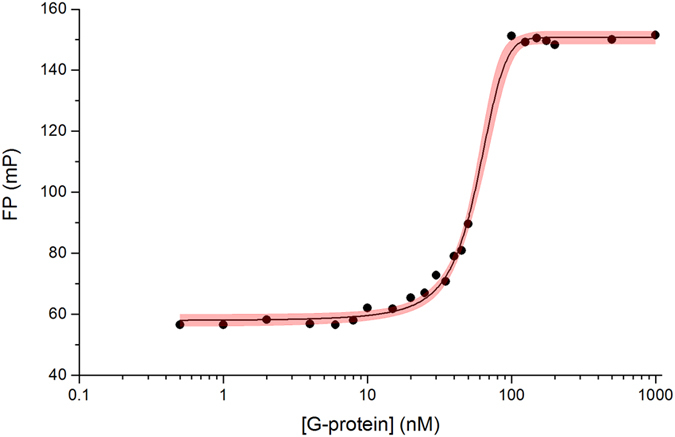
Measurement of aptamer (H8)-G protein binding by fluorescence polarization. The Alexa Fluor 488 labelled aptamer was mixed with various G-protein concentrations and the fluorescence polarization was determined. The experimental data were fitted with a 1:1 stoichiometry dose-response curve; the area highlighted with red is the 95% confidence band of the fit.

The above experiments demonstrated that the applied antibody and the selected aptamer did not compete for the same epitope but did not provide information about the viral protein target of our aptamer. In our search for the viral protein interacting partner of the selected aptamer, a recombinant, His-tagged G protein was tested as a likely candidate. Constant concentration of fluorescently labeled aptamer was incubated with various amounts of G protein and the anisotropy alteration was detected. A change of polarization was detectable at as low as 10 nM protein concentration, and 30 nM K_D_ value was calculated for the G-protein-aptamer interaction by fitting the experimental fluorescence polarization curve with a 1:1 binding model (Fig. 7).

### Feasibility of detection of diagnostically relevant RSV concentrations

**Figure 8:**
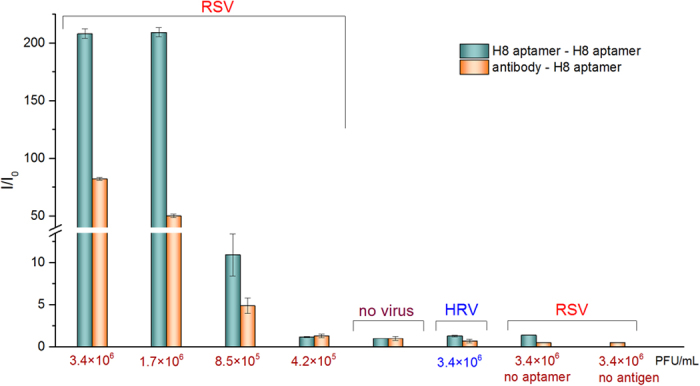
Detection of RSV at clinically relevant concentrations ALPHA. Throat swab samples were spiked with different amount of RSV and the mixtures were supplemented with biotin labeled aptamer and F protein selective antibody. Fluorescence intensities were measured following addition of streptavidin coated donor beads and protein A acceptor beads. For purely aptamer-based detection RSV, the biotin labeled aptamer was coupled to streptavidin donor and acceptor beads. The modified beads were added to throat swab samples spiked with different amount of RSV and fluorescence intensities were measured. The ratios of sample fluorescence (I) and virus-free background fluorescence (I_o_) are indicated. Both approaches provided distinctive fluorescence signal differences at clinically relevant RSV concentrations.

The mean viral loads in nasal washes of RSV infected patients are 6.12 and 4.95 log plaque forming unit equivalents (PFUe)/ml for RSV-A and RSV-B, respectively^18^. We spiked the swab samples with virus concentrations in this range to test if our most promising aptamer (H8) is suitable for detection of clinically relevant viral loads. Two types of ALPHA studies were set up: the virus binding sandwich was composed of either antibody- biotinylated aptamer or two biotinylated aptamers. In the first set up, the antibody and the aptamer were mixed with throat swab containing different amount of inactivated RSV and Rhinovirus. Following addition and incubation with Protein A acceptor and Streptavidin donor beads, the chemiluminescence of the samples was measured. There was a clear difference in signal intensity down to 8.5 × 10^5 ^PFUe/ml viral load in comparison to virus-free or Rhinovirus spiked samples (Fig. 8).

Due to the many copies of envelop composing proteins on the virus surface, RSV is theoretically detectable with a sandwich-type assay by using a single receptor. Based on this rationale, we designed a solely aptamer-dependent virus detecting approach. Prior to addition of virus including throat swab samples, the biotinylated aptamer was separately coupled to Streptavidin acceptor and donor beads. Upon addition of bead-linked aptamers to the samples with varied virus concentration, significantly higher fluorescence was detected in the RSV spiked samples (Fig. 8). Importantly, the use of solely aptamer based detection showed significantly higher sensitivity than that based on using mixed antibody-aptamer for the RSV assay.

### Stability of the RSV selective aptamer in throat swab

**Figure 9:**
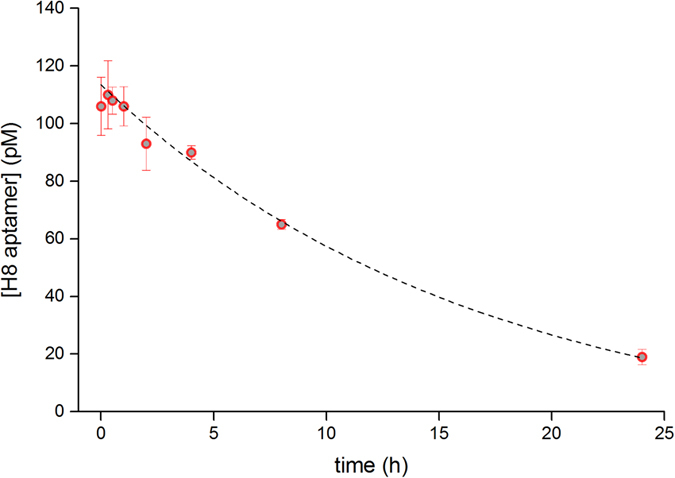
Aptamer stability analysis in throat swab samples. Throat swab samples were infused with H8 aptamer and the aptamer concentration was determined at the indicated time points by real-time PCR.

Due to their lack of 2′-OH groups, DNA molecules are resistant to 2′-endonucleases thus more stable in biological fluids than RNAs; therefore, we aimed at generating DNA aptamers for RSV detection. Nevertheless, we infused throat swab samples with 100 pM of aptamer and measured the concentration change by real-time PCR at various time points up to 24 hrs to study the stability of our selected aptamer in clinically relevant samples. The obtained data demonstrated a negligible degradation of the aptamer within the analytical time frame, i.e. the concentration of aptamer was app. 90 pM following 2 hrs incubation and dropped to 30 pM after one day (Fig. 9).

## Discussion

The first aptamer selections were described more than a quarter-century ago but so far only the therapeutic potential of aptamers has been realized^19^. Considering their relatively small size, functionality in non-physiological buffers, resistance to harsh conditions with respect to temperature and pH, on demand chemical modification, and cost-effective, constant quality production, aptamers seem to be the ideal receptors of diagnostic systems^20^. Despite the numerous advantages, only two mycotoxin detecting, ELISA-like microplate assays represent the commercially available, aptamer-dependent diagnostic systems to date^21^.

In viral diagnostics, the traditionally applied viral culture method is laborious and lacks sensitivity it became therefore obsolete and has been taken over by PCR-based approaches in most of the laboratories^22^. The viral nucleic acid detecting methods have proven to be fast, highly sensitive and specific, but they are not without shortcomings^22^. The detection of protein rather than nucleic acid components of viruses is generally technically less challenging and more suitable for rapid tests. The importance of viral protein detection is clearly indicated by the fact that two-thirds of the results reported to a RSV surveillance program were generated by rapid antigen-detection tests (RADTs)^23^.

All the presently applied antigen-detection based virus diagnostic tools depend on highly-selective antibodies. Although several aptamers have been generated for virus identification in laboratory settings, application of aptamers for viral diagnostics has not been realized, yet^10^,^24^. We demonstrated that rationally designed selection procedure could deliver aptamers, which are suitable for virus detection in clinically relevant samples. Due to the difficulties of production of purified virus, the majority of published virus selective aptamers were generated by using viral proteins as selection targets^10^. We omitted the virus purification step by immobilizing inactivated RSV onto Protein A paramagnetic beads using F protein specific antibody^2^. The immobilized virus was used as target and the stringency of selection was increased by using HRV to counter select the aptamers, which bind to the most common coinfecting virus during RSV infection. One of the most challenging tasks of the successful aptamer generation is to pinpoint the most auspicious candidates from a panel of oligonucleotides obtained by the selection procedure. We identified the most promising oligonucleotides with three different methods to ensure the applicability of selected candidates. First, we analyzed the virus binding capacity of consensus motif holding aptamers by FP, a convenient method for interaction studies. The obtained data demonstrates that the consensus motif holding aptamers can possess significantly different affinity for their target indicating the limitation of computational methods for prediction of aptamer binding capacity. Next, the traditional filter binding assay was implemented, then the obtained data were corroborated by ALPHA. A favorable feature of the presented approaches is that none of them requires chemical synthesis of the labeled oligonucleotides implying cost-effective panning of aptamer candidates. The results provided by the diverse techniques also emphasized the significance of functional testing of isolated oligonucleotides. The selection pressure is not the single factor that drives the enrichment of oligonucleotides; the polymerase reaction does not amplify all sequences equally leading to a PCR bias^25^. In accordance with this observation, we found that the strongest RSV binding aptamer was represented by a single copy amongst the sequenced oligonucleotides, while the aptamers in duplicate copies showed the least binding to the virus. To further characterize the most promising aptamer, we applied FP. These measurements demonstrated that the consensus motif holding aptamer does not compete with the monoclonal antibody of RSV vaccine but recognizes the G protein of the virus. The dissociation constant of aptamer-G protein interaction was also determined by FP and the measured low nanomolar range value positioned our aptamer amongst the virus selective aptamers with the highest affinity^10^. In our last experiments, we studied whether the selected aptamers are suitable for the detection of RSV at clinically relevant concentrations in complex protein matrices. To this end, ALPHA was applied in two arrangements; the sandwich type assay either relied on F protein selective antibody and our selected aptamer or purely on the selected aptamer. Both assay compositions showed similar sensitivity by detecting RSV at 5 log PFUe in a throat swab matrix. Although it is generally assumed that the use of aptamers is limited due to their nuclease sensitivity, it has previously been demonstrated that oligonucleotides can preserve their integrity even in a nuclease rich matrix, such as blood serum^26^. Corroborating the previous findings using blood serum, we also presented that aptamers can withstand nuclease attack up to hours in throat swab. Therefore, considering the analysis time of the presently available biomarker detecting diagnostic devices, degradation of aptamers is not expected to pose a barrier for development of aptamer based assays. Our results provide a further evidence indicating that degradation issue of aptamers is likely to be exaggerated in the scientific literature.

The highly-selective detection of the unceasingly emerging new strains is a continuous challenge for viral diagnostics that cannot be resolved by the traditional methods. The appearance of aptamers is foreseen to escalate the protein-based detection of viruses as it is indicated by the development of a panel of aptamer-dependent analytical methods for virus detection. Probably, the main bottleneck of aptamer-based viral diagnostics is the lack of aptamers, which are tailored by considering their intended application. The provided method in this study circumvents the difficulties of virus protein purification by *in situ* immobilization of target virus onto magnetic beads by using a selective antibody. The selection pressure was increased by introducing a counter-selection step with a common coinfecting virus to enhance the selectivity of isolated aptamers. Our investigations also highlight the imperative of the extended functional analysis of aptamer candidates under clinically pertinent conditions. The presented protocol resulted in an aptamer, which selectively detected RSV in complex matrices; therefore, this study provides a representative procedure for generation of virus selective aptamers with potential clinical application.

## Methods

### Virus culture, quantification and inactivation

Respiratory Syncytial Virus cultivation was performed as previously described^27^. HeLa cells were cultivated in medium (i.e. DMEM with 10% fetal calf serum (FCS) and 1% penicillin and streptomycin) to 50% confluency. The cells were infected with RSV A2 in 4 ml of medium for 2 h at 37 °C with 5% CO_2_, then 15 ml of culture medium was added to the tissue culture flasks which were incubated for 4 days at 37 °C, 5% CO_2_. Subsequently, the cells were harvested by scraping the cells using a cell scraper, the medium with the cell suspension was collected, vigorously vortexed and centrifuged for 10 min at 1800 × g to spin down the cell debris. RSV was purified by ultracentrifugation over a 30% sucrose layer for 1.5 h at 72000 × g, then aliquoted, snap-frozen in liquid nitrogen and stored at −80 °C until further use.

The virus was quantified by titration on HeLa cells followed by a permeabilization and fixation step using Perm/Fix buffers (Cell fixation and permeabilization kit from Becton Dickinson) according to the manufacturers’ protocol. The cells were incubated with FITC-labeled anti-nucleoprotein (Abcam, ab25849) for 30 min on ice washed with Perm/Wash buffers (Becton Dickinson) and measured on the flowcytometer (LSR II, Becton Dickinson) to enumerate the number of positive cells which is equivalent to infectious particles or PFUe (=plaque forming unit equivalents) expressed per volume.

Furthermore, the virus stocks were also quantified on a LightCycler 480 system for the detection of respiratory pathogens from Roche (at the clinical microbiology laboratory of the Radboud UMC).

RSV A2 was inactivated using β-propiolactone (BPL)^28^. BPL (0.025%) was added to the virus stock and incubated for 16 h at 4 °C under continuous slow shaking. Subsequently, the stock was incubated for 4 h at 37 °C to hydrolyze (inactivate) BPL and stored at −80 °C until further use.

### Throat swab collection

Throat swabs were obtained from healthy adolescents with Meus S.r.L. medical swab stick and soaked in 2 ml of PBS (1.37 M NaCl, 27 mM KCl, 20 mM KH_2_PO_4_, 100 mM Na_2_HPO_4_, pH–7.4) immediately after collection, vortexed for a while then centrifuged at 14 000 rpm for 2 min at RT. The supernatant was stored at −20 °C until the time of analysis.

### SELEX library and primers

The SELEX library was composed of 30 random nucleotides (30 N) flanked by fixed sequences: 5′-TAGGGAAGAGAAGGACATATGAT-30 N- TTGACTAGTACATGACCACTTGA-3′ (TriLink). 5′-TAGGGAAGAGAAGGACATATGAT-3′ and biotin-5′-TCAAGTGGTCATGTACTAGTCAA-3′ forward and reverse primers were used for amplification during the selection procedure. The final PCR for cloning was carried out by using the nonbiotinylated version of reverse primer. To produce fluorescently labeled aptamers, 5′-FAM labeled forward and 5′-phosphorylated reverse primers were applied In the PCR reaction mix.

### RSV immobilization onto paramagnetic beads

15 μg of F protein selective antibody (Palivizumab) was bound to 100 μl of SiMag Protein G paramagnetic bead (Chemicell) by 1 h incubation in PBS at RT. After binding of the antibody, the beads were washed with 0.2 M sodium-borate (pH 8) and the antibody was covalently linked to the ProteinG by the addition of dimethyl-pimelimidate at 0.02 M final concentration. Following 30 min incubation at RT, the bead was blocked by washing with 0.2 M ethanolamine (pH 8) and stirring in the same buffer for 2 h at RT. The antibody coated beads were stored in PBS-0.02% sodium-azide at 4 °C.

### Selection of RSV discriminating aptamers

The SELEX of RSV discriminating aptamers were obtained by four iterative cycles with gradually increasing selection pressure. Prior to use, the oligonucleotide library was heated to 95 °C for 5 min and immediately cooled on ice. First, 1 nmol of the oligonucleotide library was incubated with the antibody modified beads in selection buffer (10 μl/ml BSA, 0.1% Tween 20, 0.1 μg/ml poly(deoxyinosinic-deoxycytidylic) acid (poly(dIdC)) in PBS) to exclude the bead and antibody binding oligonucleotides. The antibody modified beads were incubated with 100 μl of 1.2 × 10^7 ^PFUe/ml RSV for 1 hour and then washed with 3 × 100 μl selection buffer. In the first selection cycle, 1 ml of the buffer was supplemented with inactivated 100 μl HRV (serotype 14) of 1 × 10^8 ^PFUe/ml (kindly provided by Kjerstin Lanke, Department of medical microbiology Radboudumc) to increase the RSV specificity of selected oligonucleotides and incubated with bead immobilized RSV for 1 h at RT with mild shaking. Then, the beads were washed three times with the selection buffer and directly used in the PCR as template without any purification. Beside the beads, the PCR mixture contained HF reaction buffer, 4 U of iProof polymerase (Biorad) 0.5–0.5 μM of forward and reverse primers, and 0.3 mM CleanAmp dNTP (Trilink). Amplification conditions were: 5 min at 95 °C, 15 cycles of 95 °C for 10 s, 63 °C for 10 s, 72 °C for 10 s and 72 °C for 1 min after the last cycle. The success of amplification was monitored on 10% polyacrylamide by gel electrophoresis. The PCR product was coupled to 50 μl streptavidin-coated paramagnetic beads (SiMAG-Streptavidin, Chemicell) for 30 min and washed with 3 × 100 μl of PBS. The nonbiotinylated strands were separated from the immobilized complementary strand by 10 min incubation with 50 μl of fresh 100 mM NaOH. The eluted ssDNA was immediately neutralized by addition of 7.5 μl of 1 M NaH_2_PO_4_. In the following rounds of selection, HRV was omitted from the buffer, and to increase the selection pressure, the incubation conditions were changed. The first three rounds of selection were performed in 1 ml of selection buffer. In round 2 and 3 the incubation time was reduced to 30 min and 20 min, respectively. In the third round an additional washing step with100 μl of 0.3 mM dextran-sulphate in PBS (pH 7.4) was also introduced. In the final selection round, the reaction volume was increased to 1.5 ml, the incubation time was 20 min and the beads were washed once with 0.3 mM dextran-sulphate solution. The PCR product of the last selection step was inserted into a cloning vector (Zero Blunt TOPO PCR Cloning Kit, Thermo Fischer Scientific) and transferred into α Select Gold Efficiency chemically competent cells (Bioline). 99 of the colonies were analyzed by colony PCR and gel electrophoresis on 3% agarose. The sequences of 96 colonies with correct length were determined by Sanger sequencing.

### Generation of labeled aptamers by PCR

Fluorescently labeled aptamers were acquired by amplifying the products of colony PCR with using 1–1 μM 5′-FAM labeled forward and 5′-biotinylated reverse primers and PCRBio mastermix (PCRBiosystems). Amplification conditions were 5 min at 95 °C, 20 cycles of 95 °C for 15 s, 55 °C for 5 s, 72 °C for 5 s, 72 °C for 1 min after the last cycle. The success of the reaction was monitored on 3% agarose by gel electrophoresis. Single stranded oligonucleotides were produced by alkali denaturation of the PCR products (see above). Biotinylated aptamers were generated following the same protocol, but the PCR mixture contained 1–1 μM of 5′-biotinylated forward primer and 5′-phosphate reverse primer. Single stranded biotinylated oligonucleotides were obtained by λ exonuclease treatment of the PCR product. To this end, the PCR products were purified by PureLink Quick Gel Extraction and PCR Purification Combo Kit (Thermo Fischer Scientific) and treated with 1 U λ exonuclease enzyme for 30 min at 37 °C (Thermo Fischer Scientific). The λ exonuclease was inactivated by heat treatment for 10 min at 80 °C.

### Filter binding assay

Prior to application, the nitrocellulose membrane (Bio-Rad) was soaked in 100 mM KOH for 10 min and rinsed with selection buffer to avoid non-specific binding of single stranded oligonucleotides. The 5′-FAM labeled oligonucleotides at 50 nM concentration were mixed with various amounts of virus in selection buffer with or without 3 times diluted throat swab. The final volume was brought up to 30 μl and the mixture was incubated for 20 min at RT. Next, the mixture was completed to 100 μl by addition of selection buffer and transferred onto the nitrocellulose membrane by using a dot-blot apparatus with 20 mbar vacuum. The membrane was washed 3 times with 100 μl selection buffer and scanned at 532 nm with Typhoon 9410 Imager. The images were analyzed with the ImageQuant software (version: 5.2).

### AlphaScreen assay

The binding assays were performed using white 384-well Optiplates (PerkinElmer) in total volume of 18 μl using Protein A acceptor and Streptavidin donor beads (PerkinElmer). Varying amount of viral samples in HBSS supplemented with BSA (1 mg/ml), poly(dIdC) (0.05 ug/ml) and EDTA (10 nM) were incubated with 10 nM final concentration aptamer and F protein selective antibody. Following 20 min incubation at RT, the acceptor and donor beads were added at 20 μg/ml final concentrations in two steps. First, the ProteinA acceptor beads were added and incubated for 1 h at RT and that was followed by the addition of Streptavidin donor beads and further 20 min incubation. The solely aptamer-dependent binding assays were done using Streptavidin AlphaLISA acceptor and Streptavidin donor beads (PerkinElmer). The aptamers were separately incubated with the acceptor and donor beads for 1 h at RT in HBSS supplemented with BSA (1 mg/ml), poly(dIdC) (0.05 ug/ml) and 10 mM ethylene glycol-bis(2-aminoethylether)-N, N, N’, N’-tetraacetic acid (EGTA). The final concentration of the aptamers and the beads were 10 nM and 20 μg/ml, respectively. After 1 h, the acceptor and the donor bead containing fractions were combined with the varying amount of viral samples and incubated for an additional hour at RT. Light signal was detected by using an EnSpire multilabel plate reader from PerkinElmer.

### Fluorescence polarization experiments

The aptamers used for FP were synthesized with a fluorescent Alexa Fluor 488 label at their 5′ (Sigma-Aldrich). RSV-G Protein (His Tag) was supplied by Hölzel Diagnostika GmbH (13029-V08H-10, manufactured by Sino Biological).

pH 8.0 Tris-EDTA buffer solution (T9285, Sigma-Aldrich) was used to prepare the aptamer stock solutions at a concentration of 100 μM. All other solutions were prepared with pH 7.4 phosphate buffered saline solution (P4417, Sigma-Aldrich).

The fluorescence intensities and fluorescence polarization values were recorded on a BioTek Synergy 2 microplate reader that uses a xenon flash lamp with a 485/20 nm bandpass filter as light source. The emitted light was passed through a 528/20 nm bandpass filter and detected with a photomultiplier tube. The microplate reader was equipped with a 510 nm dichroic mirror and a polarizer set. The FP measurements were made in a 96 well half-area flat bottom black plates (CLS3694, Corning) with each well containing 60 μl reagents. Prior to use the wells were blocked using Pierce Protein-Free (TBS) Blocking Buffer (37570, ThermoFisher Scientific). The fluorescently-labeled aptamer concentration was kept constant in all experiments at 0.5 nM. For accurate measurement of the absolute polarization the instrument specific correction factor, i.e., G-factor was determined with fluorescein solution following the instructions of the manufacturer. The polarization values were obtained from the fluorescence intensities parallel (I_||_) and perpendicular (I_⊥_) with respect to the plane of linearly polarized excitation according to the equation (1),


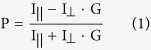


where G is the G-factor of the instrument. The end-point FP values were calculated as average of 10 measurements.

The KD of the G-protein-aptamer interaction was 30 nM as obtained from fitting the experimental fluorescence polarization curve with a 1:1 binding model.

### Aptamer stability analysis by real-time PCR

A pharyngeal swab sample was mixed with aptamer H5 in the final concentration of 100 pM in PBS and incubated at room temperature. Samples from this mixture were taken after 0 h, 0.25 h, 0.5 h, 1 h, 2 h, 4 h, 8 h and 24 h of incubation, then freezed in liquid nitrogen immediately. Next, qPCR was carried out using QuantStudio 12 K Flex PCR System. The 20 μl PCR mixture consisted of 0.8 ul of 10 μM unlabeled reverse and forward primer, 10 μl of qPCRBIO SyGreen Mix Lo-ROX (PCR Biosystems), 5 ul sample, 3.4 ul nuclease-free water. The reaction conditions of real-time qPCR were the following: initial denaturation for 5 min at 95 °C, followed by 40 cycles of denaturation for 5 s at 95 °C, annealing for 10 s at 60 °C. Melting curve analysis was performed from 60 °C to 95 °C.

### Ethics statement

Throat swabs were taken from healthy adult volunteers after obtaining verbal informed consent in the presence of the study team. Written informed consent was not obtained because sampling was non-invasive. The research ethics committee of the Radboud University Medical Centre approved the study protocol and informed consent procedure. All human sample including methods were performed in accordance with the relevant guidelines and regulations.

## Additional Information

**How to cite this article**: Percze, K. *et al*. Aptamers for respiratory syncytial virus detection. *Sci. Rep.* **7**, 42794; doi: 10.1038/srep42794 (2017).

**Publisher's note:** Springer Nature remains neutral with regard to jurisdictional claims in published maps and institutional affiliations.

## Supplementary Material

Supplementary Information
