# Supplementary material for: Aptamers for respiratory syncytial virus detection
Source: Sci Rep. 2017 Feb 21;7:42794. doi: 10.1038/srep42794 (PMC5318870; doi:10.1038/srep42794)
Supplement: Supplementary Information [file srep42794-s1.pdf]

Aptamers for respiratory syncytial virus diagnostics

Krisztina Percze, Zoltán Szakács, Éva Scholz, Judit András, Zsuzsanna Szeitner, Corné van den Kieboom, Gerben Ferwerda, Marien I. de Jonge, Róbert E. Gyurcsányi, Tamás Mészáros

Seq. ID no.	Variable region (5’ - 3’)	No. of occurrence
A1	GCAGGTAGTTTGTGATCCTTTTTTCTCAGC	1
A2	GCATGTGCAAAAAGGGGGACCCACTATCGG	1
A4	CGTCGGGTGGTCACGCGGAAAGTTGCTTTT	1
A6	TACCTCCATTGTTCTGGTAGTCAAGAAGGT	1
A7	CCTTTGCCTTTCATCTGCGGACTGATCACG	1
A8	TAAGTTAATGAATACGCCAAGAAGGCACCA	1
A9	TGCTTCAGACCCATTCTTTCATATGTCATC	1
A10	ATGTCGGGAGTAAAATGCCGCCAGTAAGTT	1
A12	GATCGATATGTCTGCCGGAATCTCTGGTGA	1
B1	TTATGATTGTGGTAATGGCGGAACTTGGGC	1
B2	CGGGAAAACGAGTGTACTTGGCAACAAACT	1
B3	TAAATGTCATAGTGATTTTAGGCATATAAA	1
**B5**	**AAGCCCCGTACCAAAGAAGGTCAGGTGTCT**	**2**
B6	TCCCACCGGTGGCTAATGCGGCCTGAGGGA	1
B7	ACGTGAAGGAGTTGGAGCTTGTGCCGCGCA	1
B9	TTACATAAGGAAAGATACTAAAGATCTTTA	1
**B10**	**TCCATATCGTTTAGCGTACGGTGGCAGTCT**	1
B11	CAGGGGAACCGCCCTTCGGTCTGTTTAAGA	1
B12	CCCCCGGCAACGAGATCCACTCAGTCCACC	1
C1	CATTTGGTGGAAGCACGCGGGTTGTGCACC	1
C2	AACTCTTACTCAACTCGCTGTGCCCCTAGG	1
C4	TGAACATTAATACCGAGCGATAGAACACAA	1
C5	CCCACGTGACGTGTTGCTTTACCATAGGCT	1
C7	CTTGGCGAGAATCAGACGGACGTTGACGTT	1
C8	CCATACTTAGATAACCTATCTTTCATAGCT	1
C9	GCAGGATTGGACCCAGTCCTACTACTGGGA	1
C10	GGACGGCGCGCACATCGAACGGCTAAGGAC	1
C11	ATCACGGGTTAAGCGACGATGAGGGGGCCG	1
C12	TACACCCAAGGGAAGTGCTTCTCAGGGTGC	1
D1	GTCAATCCAGAAGGGTTTGCGATAATGTCA	1
D2	TAAGAGAGACGCGGAGGCCCCACTCGAAGG	1
D3	TCCCCCCGCATTCCGAAGCCGATCAGGAGT	1
D4	CACCAAGATACTCATAAGGCAGTGCCGAGT	1

D6	AGGGGAAACAAATCAGTGTTGAGAGTAGGC	1
D8	AGGGAAATACCTCTATCAAATTTGGCAATC	1
D9	GGATCTTGAGTCATTAAGTACTTCGTTTGC	1
**D10**	**TAAGAGGCAGACAGTAAGAACAACTACCTT**	**2**
D11	AAGTCGGGAGAGATCAGACAGTATGCCAGT	1
**D12**	**TAAGAGGCAGACAGTAAGAACAACTACCTT**	2
E1	AGATGGCAGCATGATCGTGTAATCTGATCA	1
E2	TACGGGCCGGAACGGAAACTAGCTCAAAAC	1
E3	TAAGTAGCGCATCAAACCCCGGACAGCGAC	1
E4	CGGTCACTTATACACGAGACCAAGGATCAA	1
E5	CACTATATGCGTACAAGTGCCTGTGGCAGA	1
**E6**	**ACCCGTCGGACTCGGCCATAAATTAAAGGC**	1
E7	GGTCCGAGGACGAGATTTCTACACGTATTC	1
E8	CGGAGCGGTATAGCTTGAACCTTTCTTTCT	1
E9	TCCGAAGACCGGTAATTTCTCAATGGACTA	1
**E10**	**GTGTCCGTTCTTATTGGCGGCTCCCAATGT**	1
**E11**	**TCATTAGGTGAGTGTCCGTTCTACACTATA**	1
E12	CGCCCATATTGGATCAGGAGCCTGATCACG	1
F1	TATATGCTTGGTAATAGGCATCTTACATCT	1
F2	GTCAAATTCATTTTATGACTGCGGTTCATA	1
F3	TCGGAGTATCAAAAATGTCCAGCACCACAG	1
F4	ATCGGGAGGTGATATTAATAATAAAACCAT	1
**F6**	**AAGCCCCGTACCAAAGAAGGTCAGGTGTCT**	2
F7	CGGCAAAGAGATGTTATGTAAGTACGAGTC	1
F8	AAAAATAGACCTGCAGGCACGTATCTAATC	1
F9	ATTTAAAGGAGAGTCTACACTCGTGCATGA	1
**F10**	**ATTTAGCGTACTGCCAACCATCAGGCGCCA**	1
F11	AAGCGACAGGACCGTGGAGACCTTTCGGCA	1
F12	ACTGTAGAAATAGACTAGGATGCCGTACGG	1
G1	TGTAGTGGGCACGCAGTTCATTCCTGACAA	1
G2	TATAAACGGGGACAACCCTGGCTACTCGTA	1
G3	AAAAAGTGAAGGAGGTACGGGAAATTGTTA	1
G4	GATAGCAGGAAGTCGAGTCCACGGGTGTCC	1
G5	GTATTCACCGTTTGTTGTTATGAATGGCGC	1
G6	ACGAACAGACCGCCTCGATGTAAGCAGCCT	1
G7	AGCGCAAATTCATCACAATTGTGTATTAGG	1
G8	AGAGTGTCTCTCCATGGATTCTTTAATATT	1
G10	TATGGGATAGATCCGATAAGAAAGCTGTTA	1
G11	AAGCACCATAGTAAACCCCAAACCTAAGGG	1
G12	GATCGACGTAATCTTGTATGTAAAGAGCTT	1
H1	CACTGTCGCACGAGACTGCGGTCACATATA	1
H4	TTGGTTTTCAACCCCGCGCGCACGTAAAGC	1

**H5**	**TCGCCGTCCTGATCAGTAGCAGCCAGAACG**	**1**
H6	TGGAGCGGCGAAACACTCAGCGAAAAGGTG	1
H7	TCACCTGCTGATCTGTCTCCACTCAGGAGG	1
**H8**	**AGTGCGGTGAGCCGTCGGACATACAAATAC**	**1**
H9	AGTCAAAACTAGTGCTCTAATCATTAGGTA	1
H10	CCCGTTATCATTATAGACCTGGATAAATAA	1
H11	GTTAGGGTAACGTCGTATATGGGCAGCGTG	1
H12	GGGTTACCGTTGGCTGGTCGCCGCTCAATC	1

Supplementary Fig 1 Nucleotide sequences of isolated aptamers. Nucleotide sequences of studied aptamers are in bold.
